# Influence of Posterior Decompression for Lumbar Spinal Canal Stenosis on Intervertebral Disc Degeneration on Magnetic Resonance Imaging

**DOI:** 10.7759/cureus.84992

**Published:** 2025-05-28

**Authors:** Naosuke Kamei, Toshio Nakamae, Nobuo Adachi

**Affiliations:** 1 Department of Orthopaedic Surgery, Faculty of Medicine, University of Miyazaki, Miyazaki, JPN; 2 Department of Orthopaedic Surgery, Graduate School of Biomedical and Health Sciences, Hiroshima University, Hiroshima, JPN

**Keywords:** decompression, degeneration, disc, lumbar canal stenosis, mri

## Abstract

Introduction

Posterior decompression surgery for lumbar spinal canal stenosis (LSCS) is minimally invasive but may cause intervertebral instability and disc degeneration due to the removal of posterior support structures. This study aimed to evaluate the impact of decompression surgery on intervertebral disc degeneration using magnetic resonance imaging (MRI)-based signal ratio (SR) and disc ratio (DR) parameters.

Methods

Patients who underwent MRI three months to two years after decompression surgery for LSCS were included. Exclusion criteria included decompression at three or more levels, infections, and epidural hematomas. Sixty-three intervertebral discs from 51 patients were analyzed. SR was calculated as the ratio of the T2 signal intensity of the disc to that of the spinal cord, while DR compared the decompressed disc to the T12-L1 disc. Pre and postoperative SR and DR values were compared, and their associations with revision surgery were analyzed using multiple regression.

Results

Postoperative SR (0.402±0.225) and DR (0.642±0.299) were significantly lower than preoperative values (SR: 0.461±0.223, DR: 0.695±0.276; both *p*<0.05), indicating disc degeneration. Revision surgery occurred in 16 patients (31%), predominantly younger males. However, no significant association was found between MRI parameters and revision surgery. Regression analysis identified age (odds ratio: 0.889, *p*=0.014) and sex (female, odds ratio: 0.071, *p*=0.005) as independent factors for revision surgery. The revision surgery group had a younger mean age (67.2 ± 10.6 years) as compared to the control group (74.5 ± 7.4 years). Additionally, the proportion of males was higher in the revision surgery group (81.3%) than in the control group (45.7%).

Conclusions

Decompression surgery for LSCS resulted in short-term disc degeneration. Despite this progression, disc degeneration was not associated with the need for revision surgery. These findings suggest that early postoperative disc degeneration does not adversely affect the clinical course and therefore should not be regarded as a reason to avoid decompression surgery.

## Introduction

Lumbar spinal canal stenosis (LSCS) is one of the most common degenerative spinal conditions found in elderly people and has gained importance in the aging population in recent years [1]. This disease involves the narrowing of the spinal canal due to disc bulging and thickening of the ligamentum flavum caused by degeneration of the spinal column. As the narrowing progresses, compression of the cauda equina and nerve roots occurs, causing symptoms such as numbness and pain in the legs and back, significantly reducing the quality of daily life [2]. Treatment of LSCS varies based on symptoms and the degree of spinal deformity [3]. Posterior decompression surgery is widely used as a surgical procedure to release compressed nerves. It involves partially resecting the vertebral lamina and surgically removing the thickened ligament at the stenosis site. Recently, this surgery has evolved into a minimally invasive procedure using a microscope or endoscope [4]. However, despite being minimally invasive, it may cause intervertebral instability, as part of the posterior support structure of the spinal column is removed [5,6]. Reported reoperation rates after decompression alone surgery vary considerably across studies, influenced by the duration of follow-up and type of decompression surgeries. Overall, these rates have been reported to range from 1.6% to 41.3% over follow-up periods spanning from 3 months to 17.7 years. Reoperations following decompression alone surgery are predominantly attributed to pathological conditions occurring at the same spinal segment as the initial procedure, such as disc herniation and restenosis. These account for approximately 52.1% to 100% of all reported reoperation cases [7]. Disc herniation and restenosis after posterior decompression surgery may be due to progressive degeneration of the spine caused by the resection of posterior supportive structures [8]. Therefore, in this study, disc degeneration was evaluated as an indicator of progressive spinal degeneration after posterior decompression surgery. We previously reported that disc degeneration can be evaluated using the T2 signal ratio (SR), which is the ratio of the T2 value in the central portion of the disc to the T2 value in the conus medullaris of the spinal cord on magnetic resonance imaging (MRI) sagittal section [9]. This study aimed to quantitatively evaluate disc degeneration in the short term after decompression surgery for LSCS using MRI T2 SR and to clarify its association with revision surgery.

This article was previously presented as a meeting abstract at the 39th Annual Research Meeting of the Japanese Orthopaedic Association (JOA) on October 17, 2024.

## Materials and methods

Patients

This retrospective case-control study included patients who underwent posterior decompression surgery for lumbar spinal canal stenosis (LSCS) at our hospital between January 1, 2020, and December 31, 2022. Eligible patients were those who had lumbar MRI scans performed preoperatively and again between three months and two years postoperatively. Patients were included if they underwent decompression at one or two lumbar levels. Those who had decompression at three or more levels, developed postoperative infections or epidural hematomas, or lacked appropriate imaging follow-up were excluded. Epidural hematomas were detected by postoperative MRI, and early reoperation for hematoma removal was performed in all such patients. These patients were excluded to eliminate confounding effects on postoperative image evaluation. Patients who underwent revision surgery after the initial decompression were assigned to the revision surgery group. Those who were followed for more than one year postoperatively without requiring reoperation were assigned to the control group. Lumbar canal decompression surgery was performed under a microscope using a spinous process-splitting approach, preserving the facet joints [10,11]. Finally, 51 patients were included in this study. Preoperative and postoperative MRI midsagittal T2-weighted images were used to evaluate the SR of the discs at the same level as the decompression surgery. MRIs were obtained using MR scanners (Ingenia 3.0T, Philips, Amsterdam, Netherlands; Ingenia 3.0T CX Quasar Dual, Philips; Vantage Titan 3T, Canon Medical Systems, Ohtawara, Japan; TTRILLIUM Oval 3T, FUJIFILM Healthcare Systems, Tokyo, Japan). The T2-weighted images were obtained using a fast spin-echo technique. The T2-weighted imaging parameters were as follows: TR: 3000-4500 ms, TE: 90-110 ms, echo train length: 15-25, flip angle: 90 degrees, refocusing flip angle: 120-140 degrees, matrix: 320 × 480 (512 reconstruction), field of view: 250-300 mm, slice thickness: 4 mm, number of excitations: 1-2, and the scan times were 2 minutes 18 seconds to 2 minutes 38 seconds.

MRI assessments

**Figure 1:**
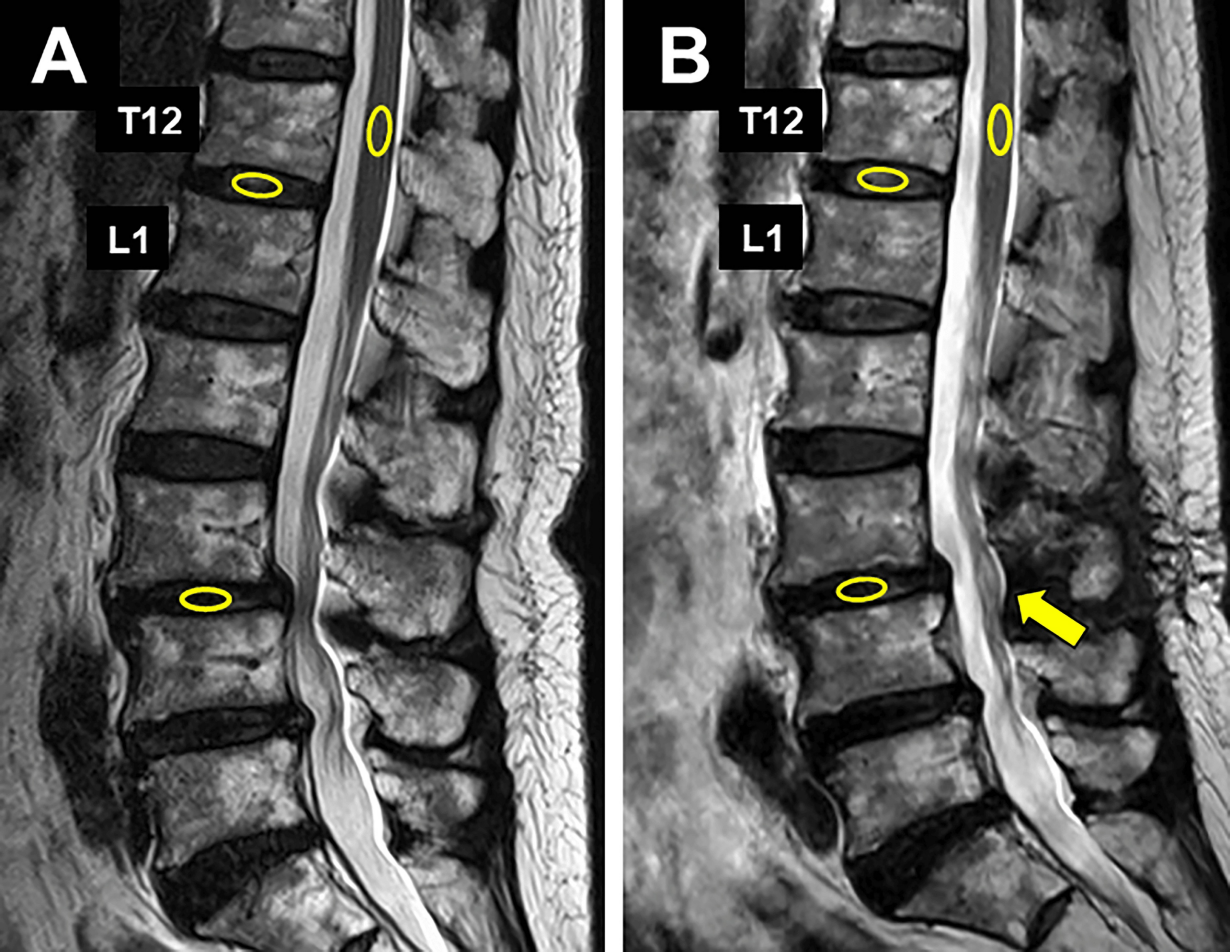
Area where the T2 signal intensities were measured Yellow ellipses on the midsagittal T2-weighted images indicate regions of interest at the center of the disc (T12-L1, decompression level) and in the conus medullaris of the spinal cord at the T12 vertebral level. The yellow arrow indicates the site where posterior decompression was performed. Preoperative MRI (A), postoperative MRI (B)

The center of the intervertebral disc and conus medullaris of the spinal cord at the T12 level were designated as the region of interest, and the signal intensity (SI) values were obtained using ShadeQuest/ViewR-DG software (Fujifilm, Tokyo, Japan) (Figure 1).

To reduce image bias, a region of interest of at least 50 pixels was selected for each point. A spine surgeon with more than 10 years of experience (NK and TN) evaluated each patient's MRI, and the average of the two was used for the analysis. The observers were blinded to patient data. SR was calculated using the SI values of the intervertebral disc (SI disc) and spinal cord (SI cord) according to the following formula [9].

$SR = \frac{SI_{\text{disc}}}{SI_{\text{cord}}}$

In addition, the ratio of the SI of the disc at the surgical site to the SI of the T12-L1 disc was evaluated as the disc ratio (DR) to distinguish between disc degeneration due to natural processes and the influence of decompression surgery.

Statistical analysis

Continuous variables are expressed as mean values ± standard deviation. Statistical analysis was performed using SPSS version 22.0 (IBM Corp., Armonk, NY, USA) and JMP® 17 (SAS Institute Inc., Cary, NC, USA). Inter-rater reliability of SR was assessed using the intra-class correlation coefficient (2,1). Intra-class correlation coefficient <0.50 was defined as low reliability; 0.50-0.75, moderate reliability; 0.75-0.89, good reliability; and ≥0.90, excellent reliability [12]. The Wilcoxon signed-rank test was used for preoperative and postoperative comparisons of SR and DR. The difference between the preoperative and postoperative values of SR and DR was evaluated as the ratio of the postoperative value to the preoperative value (change in SR, change in DR). The correlation between the MRI parameters (SR, DR, and their postoperative rates of change) and age or the number of days between surgery and the second MRI scan was evaluated using Pearson's product rate correlation coefficient. Patients who underwent revision surgery due to spinal canal restenosis and those who did not undergo revision surgery were compared for age, sex, disc level with decompression, rate of two-level decompression, preoperative SR and DR, changes in SR and DR, and the number of days between surgery and the second MRI scan. The Wilcoxon rank-sum test was used to compare continuous variables, and Fisher's exact test was used to compare nominal variables. The association between revision surgery and these factors was analyzed using logistic regression analysis.

## Results

Demographic characteristics

The mean age of total patients was 72.2±9.1 years, with 29 male and 22 female patients. Decompression surgery was performed at 1 site in 39 patients and at 2 sites in 12 patients, resulting in a total of 63 evaluated discs. The mean time from decompression surgery to the second MRI scan was 314.9±153.7 days (Table 1).

**Table 1:** Demographic data of patients (n = 51) The numbers shown in the sex and number of decompressions are the number of patients. SD: standard deviation

Variables	Mean ± SD	Range
Age (years)	72.2 ± 9.1	50-88
Time from decompression surgery (days)	314.9 ± 153.7	94-619
Variables	Frequency	Percentage
Sex		
Male	29	56.90%
Female	22	43.10%
Number of Decompressions		
One site	39	76.50%
Two sites	12	23.50%

MRI parameters

The inter-rater reliabilities for both SR and DR in the preoperative and postoperative periods were excellent Specifically, the ICC for preoperative SR was 0.996 (95% confidence interval (CI): 0.992-0.998, *p*<0.001), and for postoperative SR, it was 0.988 (95% CI: 0.976-0.993, *p*<0.001). Similarly, the ICCs for preoperative and postoperative DR were 0.989 (95% CI: 0.982-0.993, *p*<0.001) and 0.992 (95% CI: 0.986-0.995, *p*<0.001), respectively. In the analysis including all patients, the preoperative SR was 0.461±0.223 and the postoperative SR was 0.402±0.225, with the postoperative value being significantly lower than the preoperative value (*p*<0.001). The preoperative DR was 0.695±0.276, and the postoperative DR was 0.642±0.299. The postoperative DR was also significantly lower than the preoperative values (*p*=0.003). Significant differences were not found in the MRI parameters (SR, DR, and their postoperative rates of change) according to sex (*p*=0.288 for preoperative SR, *p*=0.087 for postoperative SR, *p*=0.486 for change in SR, *p*=0.086 for preoperative DR, *p*=0.150 for postoperative DR, *p*=0.995 for change in DR). No significant correlation was observed between MRI parameters and age (Table 2). In addition, no significant correlation was noted between the number of days between surgery and the second MRI scan and the MRI parameters (Table 3). MRI parameters were also compared between the two groups based on the number of days between surgery and the second MRI scan (<1 year, n=37 vs ≥1 year, n=26), but no significant differences were found in any of the parameters (*p*=0.796 for postoperative SR, *p*=0.133 for change in SR, *p*=0.402 for postoperative DR, *p*=0.238 for change in DR).

**Table 2:** Correlation between MRI parameters and age Pearson's product rate correlation coefficient was used for statistical analysis.

Variables	Correlation coefficient	*p* value
Preoperative SR	-0.198	0.121
Postoperative SR	-0.185	0.147
Change in SR (%)	-0.017	0.896
Preoperative DR	-0.052	0.683
Postoperative DR	0.026	0.841
Change in DR (%)	0.147	0.251

**Table 3:** Correlation between MRI parameters and the number of days between surgery and the second MRI scan Pearson's product rate correlation coefficient was used for statistical analysis.

Variables	Correlation coefficient	*p* value
Postoperative SR	0.087	0.498
Change in SR (%)	0.181	0.155
Postoperative DR	0.130	0.309
Change in DR (%)	0.176	0.168

Factors associated with revision surgery

A comparison of patients who underwent revision surgery versus those who did not for each parameter (age, sex, level with decompression, rate of two-level decompression, MRI parameters, and number of days between surgery and the second MRI scan) is summarized in Table 4. Patients who underwent revision surgery were significantly younger (*p*=0.023) and more frequently male (*p*=0.031). No significant differences were found between patients who underwent revision surgery and those who did not undergo revision surgery in any other factor. In the control group, the postoperative SR and DR were significantly lower than the preoperative values, whereas in the revision surgery group, no significant differences were noted between the preoperative and postoperative values. In addition, multiple regression analysis was used to evaluate the association between several factors (age, sex, change in SR, and change in DR) and revision surgery (Table 5). Multiple regression analysis showed that only age and sex were significantly associated with revision surgery, with younger age (odds ratio 0.889, *p*=0.006) and fewer women (odds ratio 0.071, *p*=0.006) among patients who underwent revision surgery. No significant association was observed between the MRI parameters (change in SR and change in DR) and revision surgery.

**Table 4:** Comparison of patients with and without revision surgery Data are shown as mean ± standard deviation for continuous variables. Fisher's exact test was used for the statistical comparison of the number of patients per level with decompression, while the Wilcoxon rank-sum test was used for all other statistical comparisons. * Significantly lower than preoperative values (p<0.05).

Variables	Control	Revision surgery	p-value
Number of patients	35	16	
Number of discs	45	18	
Age	74.5±7.4	67.2±10.6	0.023
Sex			
Male	16 (45.7%)	13 (81.3%)	0.031
Female	19 (54.3%)	3 (18.8%)	
Number of patients per level with decompression			
L1-2	1	0	0.78
L2-3	5	1	
L3-4	18	7	
L4-5	18	10	
L5-S	3	0	
Rate of 2-level decompression	10 (28.6%)	2 (12.5%)	0.296
Preoperative SR	0.471±0.261	0.438±0.149	0.897
Postoperative SR	0.403±0.244*	0.399±0.176	0.558
Change in SR (%)	87.7±25.0	91.8±20.8	0.665
Preoperative DR	0.682±0.285	0.729±0.257	0.589
Postoperative DR	0.625±0.294*	0.684±0.314	0.479
Change in DR (%)	92.6±21.2	93.5±21.8	0.885
Number of days between surgery and MRI retest	314.0±152.1	317.0±162.3	0.819

**Table 5:** Multiple regression analysis of factors associated with revision surgery Logistic regression analysis was used for statistical analysis.

Variables		Odds ratio	95%CI	p-value
Age		0.889	0.825-0.971	0.006
Sex	Male	14.010	2.189-89.652	0.006
	Female	0.071	0.011-0.456	
Change in SR (%)		1.099	0.004-398.647	0.974
Change in DR (%)		1.536	0.003-648.021	0.888

## Discussion

This study demonstrated that posterior decompression surgery for LSCS resulted in measurable short-term intervertebral disc degeneration at the decompression level. The significant reduction in both SR and DR postoperatively indicates that degeneration may progress even after minimally invasive procedures. Despite these findings, no direct association was observed between postoperative disc degeneration and the need for revision surgery. These results suggest that while structural changes in the intervertebral disc can occur shortly after surgery, they may not directly translate to clinical outcomes requiring additional surgical intervention.

Only a few studies of disc degeneration after posterior decompression surgery for lumbar spinal canal stenosis have been reported [13,14]. Nakajima et al. evaluated disc degeneration from 2 to 11 years postoperatively for the progression of disc wedging and the decrease in disc height and reported their association with preoperative vertebral bone marrow edema [13]. However, they did not evaluate changes in SI on MRI of the intervertebral disc, as we did. Fujii et al. reported that in 62 of 258 patients who underwent posterior decompression for lumbar spinal canal stenosis and had an MRI 10 years after surgery, disc degeneration progressed more than in healthy controls but was not associated with low back pain scores [14]. They evaluated disc degeneration using a scoring system that added posterior disc protrusion and disc space narrowing to the T2 SI classification using modified Pfirrman grading [15]. Both studies evaluated disc degeneration over a long-term postoperative period. In addition, they evaluated disc degeneration in terms of stepwise changes in disc SI on MRI and changes in disc height, which are not suitable for capturing subtle changes in disc degeneration. The mean time between the initial surgery and the date of MRI for revision surgery in the current study was 317 days. Therefore, to analyze the association between reoperation and disc degeneration, assessment of disc degeneration in a shorter postoperative period than previously reported was necessary, and a quantitative evaluation that could capture even subtle changes in disc degeneration was needed. Previous studies could only detect obvious progression of disc degeneration, with changes observed over several years, whereas our study was able to detect subtle changes detectable within two years using MRI-based quantitative ratios (SR and DR).

In the analysis of all patients in the present study, both SR and DR values were significantly lower postoperatively than preoperatively. This finding indicates that degeneration progressed in a relatively short period of time (314.9±153.7 days) in discs at the decompression level. Our previous study reported a significant correlation between the SR and the Pfirrmann grade of the intervertebral disc [9,15]. In the present study, both preoperative and postoperative SR values corresponded with Pfirrmann grades III (L4-5 disc, SR value 0.73±0.16) and IV (L4-5 disc, SR value 0.37±0.14). The difference in preoperative and postoperative SR values was not sufficiently large to change the Pfirrmann grade by one level. Our approach using SR and DR provides a more quantitative assessment of subtle changes. Compared to traditional methods, such as Pfirrmann grading, the T2 signal intensity ratios allow for the earlier detection of degeneration and provide additional insight into the progression of postoperative changes.

The analysis of all patients in the present study showed no significant effect of age, sex, or the time between surgery and the second MRI scan on the MRI parameters. In the comparison of patients with and those without revision surgery, the only significant differences were in age and sex, with no significant differences in MRI parameters. Multiple regression analysis showed that only age and sex were significantly associated with revision surgery. Previous studies reported on the risk factors for revision surgery after decompression of lumbar spinal canal stenosis [16-18]. Although no studies have analyzed conditions identical to ours, the risk factors for revision surgery, comprising decompression and fusion for lumbar spinal stenosis, included males, individuals in their 60s, alcohol consumption, smoking, obesity, and diabetes mellitus [16,17]. In addition, the presence of lumbosacral transitional vertebrae, facet degeneration, and paraspinal muscle atrophy was identified as a risk factor for revision surgery after endoscopic decompression [18]. In the present study, men were at a higher risk of revision surgery, and the mean age of the revision surgery group was in the 60s, which is consistent with previous reports. The changes in SR and DR after decompression surgery in this study suggest short-term progression of disc degeneration. However, no association was found between this progression and an increased risk of revision surgery. Therefore, the progression of disc degeneration after surgery does not affect clinical outcomes and should not be a reason to avoid decompression surgery.

Previous quantitative evaluations of intervertebral disc degeneration using MRI have required specialized imaging conditions, including T1 rho, T2 mapping, T2* mapping, ultra-short time-to-echo, delayed gadolinium-enhanced MRI of cartilage, diffusion-weighted imaging, glycosaminoglycan chemical exchange saturation transfer imaging, and dynamic contrast-enhanced MRI [19-30]. However, the evaluation using the signal intensity ratio used in this study does not require specialized sequences, making it more accessible in routine clinical settings. In addition, a previous study demonstrated a significant correlation between SR and Pfirrmann grades, suggesting that SR reflects disc degeneration, and both a previous study and this study demonstrated high reproducibility of SR [9].

One possible mechanism underlying this early postoperative disc degeneration is the biomechanical alteration caused by decompression surgery. Removal of posterior elements may lead to altered stress distribution on the intervertebral disc, especially in segments already compromised by stenosis. This change may accelerate mechanical stress on the annulus fibrosus and nucleus pulposus dehydration, leading to measurable degeneration within a short period. Although this degeneration may not cause immediate symptoms, it could potentially predispose the segment to later instability or further deterioration, suggesting the need for long-term follow-up in such patients.

This study had several limitations. Due to the retrospective design of this study, potential selection and information biases cannot be entirely ruled out. To mitigate these biases, patients were selected consecutively, and consistent inclusion and exclusion criteria were applied. However, the retrospective nature of the study inherently limits the ability to control for confounding variables and may introduce unrecognized biases in patient selection and outcome assessment. Future prospective studies with non-surgical control groups are warranted to validate these findings in a broader clinical context. No device calibration or image normalization, such as phantom-based correction, was performed. To minimize device-to-device variability, signal strength ratios (SR, DR) were calculated rather than absolute values. In addition, all imaging was reviewed blindly by two independent raters. The absence of a radiologist may be considered a limitation. However, excellent inter-rater reliability supports the validity of the evaluations in this study. The timing of decompression surgery differed between the control and reoperation groups, and the rate of revision surgery is unknown. Patients who underwent revision surgery always had an MRI evaluation before the procedure. However, only a subset of patients who experienced a good postoperative course after decompression surgery consented to a second MRI evaluation. Therefore, the association between revision surgery and MRI parameters within a consecutive patient population could not be analyzed. In this study, there was no control group consisting of asymptomatic patients or patients who did not undergo surgical treatment. Because it is rare to perform multiple lumbar spine MRI studies in a short period of time on asymptomatic or conservatively treated patients, it was not possible to perform a controlled study with these patients as the control group. Smoking, obesity, diabetes, and clinical symptoms were not assessed in this study because they were not recorded routinely in a standardized format, and imaging evaluations were limited to MRI. The purpose of this study was not to identify the factors associated with revision surgery but to determine the influence of decompression surgery on disc degeneration. This study did not evaluate differences in surgical skills among surgeons. The short-term follow-up in this study may not capture long-term results. In addition, since this study is retrospective, sample size calculations were not performed in advance, and the sample size may have been small.

## Conclusions

Decompression surgery for lumbar spinal canal stenosis leads to progressive intervertebral disc degeneration at the decompression site postoperatively. However, the degree of degeneration was not associated with an increased likelihood of revision surgery. Younger age and male sex were the primary factors associated with reoperation, suggesting that clinical outcomes are influenced more by patient characteristics than by early structural changes in the disc. These findings underline the need for personalized postoperative care and highlight the limited clinical impact of early postoperative disc degeneration in most cases.
